# Minutes of the Kentucky State Dental Society

**Published:** 1880-08

**Authors:** 


### MINUTES OF THE KENTUCKY STATE DENTAL SOCIETY.

*Held in Richmond, June* 1, 2 *and* 3, 1880.

The tenth annual meeting of the Kentucky State Dental Association, held in the hall of Richmond Commandery, K. T., No. 19, at Richmond, Ky., June 1, 1880.

The Association was called to order at 5 p. m. by the President, Dr. J. S. Cassidy, of Covington. After prayer by Prof. L. G. Barbour, of Central University, the following officers and members answered to their names:

J. S. CASSIDY, President.
J. HOOPER, Vice President.
C. G. EDWARDS, Secretary.
J. F. CANINE, Treasurer.
W. H. GODDARD,
F. PEABODY,
A. O. RAWLS,
A. W. SMITH,
J. B. KIDD,
C. E. DUNN,
P. S. DEDMAN,
J. TAFT, and
H. A. SMITH, *Hon. Members*, being present. The minutes of the last annual meeting was called for and a portion read, on motion, the further reading was dispensed with and the minutes as printed were adopted.

Dr. Peabody introduced the subject of members who had been suspended for arrearage. After some discussion, he offered the following resolution :

*Resolved*, That all delinquent members now present be reinstated on payment of their arrearage.

Subject discussed and laid over.

Adjourned to meet at 8 p. m.

TUESDAY EVENING, JUNE 1.

The Association was called to order by the President, at 8 p. m.

The author of the resolution before the house at time of adjournment of afternoon session being absent, on motion, it was postponed until his return.

On motion of Dr. Goddard, physicians and others of Richmond were invited to the sessions of the Association.

The Executive Committee reported the hours of meeting for the session as follows: from 8:30 a. m. to 12 m.; from 2 to 5:30 p. m.; evening session as agreed upon.

A letter of resignation from Dr. Doyle being read by the Secretary, on motion, it was accepted.

The Board of Censors recommending Dr. E. W. Ruth, of Maysville, he was unanimously elected an active member of the Association.

Dr. Peabody's resolution was called up, discussed and carried, and delinquent members present were reinstated.

Dr. Dunn's amendment to By-Laws 4 and 5, as offered at Louisville, at the ninth annual meeting, was called up and unanimously carried.

On motion of Dr. Goddard, one dollar was donated to each member who had paid $3 00 annual dues, and to Dr. Ruth $2.00, who had paid $3.00 admission fee and $3 00 annual dues.

Dr. Van Antwerp paid $8.00 arrearage and was reinstated. On motion of Dr. Goddard, Dr. Van Antwerp was donated the sum of $6.00.

On motion of Dr. Peabody, the President appointed the following committee to act upon the death of Dr. S. S. White: Drs. Peabody, Goddard and Taft.

On motion of Dr. Dunn, the President appointed a committee of three, consisting of Drs. Peabody, Smith and Goddard, to request the opinion of a lawyer as to the legality of the organization of this Association.

Dr. J. A. McIntyre, of Carlisle, made application for membership. On being recommended by Board of Censors, he was elected an active member of the Association.

Dr. A. W. Smith extended an invitation from the Hon. Cassius M. Clay, to visit his house in a body and accept his hospitality, on Thursday, at 3 p. m. On motion, the invitation was unanimously accepted.

Dr. Smith also communicated the following invitations: an invitation from Prof. Fairchild, of Berea College, to visit that Institution in a body. An invitation from Prof. Breck, Chancellor of Central University, to visit his institution in a body; and similar invitations from Profs. Barbour and Tobin, to visit their departments of the university.

On motion of Dr. Taft, the Association accepted the invitations to visit the Institutions as far as possible, to-morrow afternoon.

Dr. Smith extended a cordial invitation to the members of Association to accept his hospitalities at 8 o'clock to-morrow, Wednesday Evening.

On motion, Dr. Smith's invitation was cordially accepted.

The committee reported on the death of Dr. S. S. White as follows:

"It is always sad to contemplate the death of a friend, doubly so, when we know the fact that our friend is dead. In the death of Dr. S. S. White each member of our profession has lost a friend, one who was ever ready not only to give his talents, but his money, in any way by which the dental profession could be advanced, and his aim was to elevate the branch in which he was so deeply interested, to the highest pinnacle of excellence and beauty, much of which he did accomplish. Those who knew him best loved him most. But alas! in the meridian of life, away from the fond endearments of home, in a foreign land whence he had gone to seek new vigor and health, among strangers, he was called to cross that 'mysterious sea whose waves have never borne the image of a returning sail.' He has passed on and upward to 'the undiscovered country from whose bourne no traveler returns.' Henceforth his place is vacant; we shall see him here below no more. We must soon follow. May it be our aim to do our duty, and if we can benefit mankind a tithe what he has done, we will have accomplished a noble work.

"*Resolved*, That the Kentucky State Dental Association has heard with deep regret the death of Dr. S. S. White, and hereby records its testimony of him as an honorable man and upright citizen; and also convey to his estimable and bereaved family its sincere condolence.

"*Resolved*, That the Association forward a copy of the above under its seal to his family, and record the same upon its books."

F. PEABODY,<br>W. H. GODDARD, } *Com.*<br>J. TAFT.

On motion, the report of the committee was unanimously adopted.

Adjourned until to-morrow, 8:30 a. m.

WEDNESDAY.

Association met and was called to order by the President, at 8:30 a. m.

Minutes of the previous meeting were read and adopted.

On motion of Dr. Goddard, Dr. Dunn was requested to report the discussions of the Association.

The Board of Censors recommended Dr. M. L. S. Buckner, of Shelbyville, for membership. Dr. Buckner was unanimously elected an active member of the Association.

Dr. R. C. Morgan, having paid his delinquent dues, amounting to $8, was reinstated. On motion, the sum of $6 was donated to Dr. Morgan.

The President, Dr. J. S. Cassidy, then delivered his address, which was heard with pleasure and marked attention, and was referred to a committee, composed of Dr. Rawls, Peabody and

Dedman, to select such portions as may be desirable for the Association to consider.

On motion of Drs. Rawls, Dr. Cassidy was tendered a vote of thanks for his able and interesting address.

The committee on the President's address reported, calling special attention to that part referring to dental education, pathology and therapeutics.

The Treasurer's report being called for, Dr. Canine proceeded to make his report. On motion, the report was referred to the Finance Committee, who reported as follows:

| | | |
|---|---|---|
| 1880. | Balance on hand June, 1879 | $105 60 |
| | Received during the year | 51 00 |
| | | $156 60 |
| | Current expenses during the year | 44 13 |
| | Balance June 1, 1880 | $112 47 |

Examined and found correct. Report adopted.

The subjects for discussion being called up, the first subject on the programme being anatomy, the committee not being ready to report, Dr. Rawls opened the discussion and was followed by Dr. Taft.

Subject passed. The next subject, physiology, was passed temporarily, and pathology taken up and discussed by Drs. Peabody, Rawls and Van Antwerp.

The Board of Censors presented the name of Dr. F. M. Miller, who was unanimously elected an active member of the Association. Letters were read from Drs. L. W. Wells, of Paducah and Geo. W. Keely, of Ohio, and ordered to be filed. The subject of pathology was renewed, and discussed by Drs. Taft, Peabody, A. W. Smith, H. A. Smith, Canine, and P. C. Sutphin, M. D., a practicing physician of Hardysville. Subject temporarily passed.

On motion of A. W. Smith, the Association decided to accept the invitation of the Profs. of Central University to visit that institution at 5 p. m.

Bill of the Secretary was reported correct and ordered to be paid.

Adjourned until 2 p. m.

### WEDNESDAY AFTERNOON.

Association called to order by the President at 2 p. m. Minutes of Morning Session read by the Secretary and approved.

Pathology was continued and discussed by Drs. Taft, A. W. Smith, Canine, Dedman, Rawls, H. A. Smith, Peabody, and Justice. Subject passed.

The committee appointed to inquire into the legality of the Association reported as follows :

" *To the President and Members of the Kentucky State Dental Association.*

"Your Committee would report that they have consulted the Hon. Gov. McCrary in regard to the legality of the organization of this Association, and he states that from the record he would consider there was no doubt of its legal condition. He would further state, that be the organization legal or otherwise, it would in no way interfere with the law as it stands. The law now states that no one can practice in the State in our profession unless he possesses a diploma from a recognized college, or has passed a satisfactory examination before the Board of Examiners appointed by the Association. In the latter case, they may give him a certificate to practice. If the Association is not legally organized, they simply have not power to give the applicant for practice a certificate, and they must be graduates. The law as passed is a law, whether there is an association or not."

[Signed], Respectfully, F. PEABODY,
W. H. GODDARD, } *Com.*
A. WILKS SMITH.

The report was submitted and adopted.

Adjourned until 8:30 Thursday morning.

### THURSDAY MORNING.

The Association was called to order by the President at 8:30.

The minutes of previous meeting being read and accepted, the Association proceeded to business. Bills of Committee of Arrangements approved and ordered paid.

Dr. Goddard offered the following resolution :

"*Resolved,* That the thanks of the members of the Kentucky State Dental Association be and are hereby tendered to the citizens of Richmond, Ky., who have so cordially extended such a hearty welcome to us all, and whose hospitality has never been equaled at any place where we have heretofore held our meetings, the recollection of which will be ever cherished in our memories."

The resolution was unanimously adopted and ordered to be published in the Richmond papers.

On motion of Dr. Goddard, the Association authorized the Treasurer to pay the sum of ten dollars to Dr. Dunn for his services as reporter for the Association.

Dr. Rawls offered the following resolution, which was unanimously adopted:

"WHEREAS, The States of Ohio, Indiana and Kentucky all having a law appointing a Board of Examiners, to examine all persons entering the practice of dentistry in those States, therefore be it

"*Resolved*, That the State Board of Examiners of this Association are hereby requested or authorized, to recognize the certficates issued by the Board of Examiners of Ohio and Indiana, as sufficient evidence of the character and qualifications of the one receiving the same, to practice dentistry in this State, and may issue their certificate on such evidence, according to the laws governing the same."

The subjects of chemistry and physiology were called and passed, and therapeutics taken up and discussed by Drs. Rawls, Peabody, Van Antwerp, Edwards, Cassidy, and Sutphin. Subject passed.

On motion, the hour of 12 m. was decided on for the election of officers for the ensuing year.

Dr. A. W. Smith opened the discussion on Operative Dentistry, and was followed by Drs. Canine, Taft, Edwards, and Peabody.

Trof. Tobin, of Central University, was introduced to the Association, and entertained the members with some interesting remarks as to the possible chemical action of lead as a filling on root canals.

The hour appointed for the election of officers having arrived, the Association proceeded to ballot, with the following result:

C. G. Edwards, Louisville, President; Wm. Van Antwerp, Mt. Sterling, Vice President; W. W. Justice, Winchester, Secretary; J. F. Canine, Louisville, Treasurer.

EXECUTIVE COMMITTEE.

W. W. Justice retiring, A. O. Rawls was elected for three years.

BOARD OF CENSORS.

J. Hooper's term expiring, he was re-elected.

BOARD OF EXAMINERS.

C. E. Dunn was re-elected.

Drs. Peabody, Miller, Morgan and Dedman were elected delegates to the American Dental Association. Lexington, Ky., was chosen as the place for next meeting.

On resolution offered by Dr. Dunn, the President appointed Drs. Dunn, Rawls, and Van Antwerp a committee to draft resolutions on the death of Dr. Shadoan.

Having accepted an invitation to visit the Hon. Cassius M. Clay, this afternoon, the Association adjourned until 8 p. m.

THURSDAY EVENING.

The Association was called to order to order by the President at 8:30 p. m.

The officers elected were then escorted to the Chair by Drs. Ruth and Buckner, and regularly installed.

The committee appointed to draft resolutions on the death of Dr. W. H. Shadoan, reported as follows:

"WHEREAS, Dr. W. H. Shadoan, one of the charter members of this Association, and the first Secretaty of the same, in November last was called by an All-Wise Providence to pass that bourne from which no traveler returns; and,

"WHEREAS, Dr. Shadoan was an active and earnest worker in the interests of this Association in the early days of its organization, therefore be it

"*Resolved*, That the dental profession, by the death of Dr. Shadoan, has lost one who for many years labored in its cause—one who loved and endeavored to advance its interests.

"*Resolved*, That a memorial page in our record-book be set apart and inscribed to his memory.

"*Resolved*, That a copy of these resolutions be sent to the family of deceased."

[Signed], C. E. DUNN, A. O. RAWLS, WM. VAN ANTWERP, } *Com.*

Dr. W. Smith, chairman of committee appointed in 1878 to prepare a suitable treatise on the care of the teeth, to be published and used by the members of this Association, stated that they had met with difficulties which had not been anticipated, owing to the members not being able to confer together. The committee was granted further time.

Regular business was then taken up, and the subject of Operative Dentistry was passed. Mechanical Dentistry was also passed.

On motion of Dr. Goddard, it was directed that a copy of the DENTAL REGISTER, containing the proceedings of this meeting, be sent by the editor to each member of this Association, at the expense, if any, of the Association.

The Executive Committee reported the following subjects and the committees thereon, for our next annual meeting:

Anatomy—Wm. Van Antwerp.
Physiology—A. W. Smith.
Histology— ———
Pathology—A. O. Rawls.
Therapeutics—F. Peabody.
Operative Dentistry—J. Hooper, J. B. Kidd.
Mechanical Dentistry—J. F. Canine, E. W. Ruth.
Miscellaneous—C. L. Dunn, P. T. Dedman.

Dr. A. W. Smith made the following motion, which was seconded by Dr. Rawls: That the Association offer a prize of $25.00 for the best essay on disease of the antrum. Discussed, and by motion was laid on the table.

Dr. Rawls then moved that this Association offer a prize of $25 for the best essay on disease and treatment of the antrum, and a committee of three be appointed by the President to examine and decide upon the merits of such essays as may be presented. Dr. Peabody offered the following resolution as an amendment:

"*Resolved,* That this Association offer a prize of $25 for the best essay on the subject of disease and treatment of the antrum, provided, the committee to decide, consider the essay worthy of a prize. That a committee of three be appointed by the President for the purpose of deciding as to the best essay offered, contending for the prize of $25, and that the committee be appointed at the next meeting." The amendment was accepted and carried.

It was moved and seconded that a vote of thanks of this Association be tendered the Richmond Commandary for the gratuitous use of this hall. Carried.

A vote of thanks was also tendered to Dr. Smith for his able exertions in behalf of this Association.

The Association adjourned to meet at Lexington, on the first Tuesday in June, 1881.

